# CenterNet-Saccade: Enhancing Sonar Object Detection with Lightweight Global Feature Extraction

**DOI:** 10.3390/s24020665

**Published:** 2024-01-20

**Authors:** Wenling Wang, Qiaoxin Zhang, Zhisheng Qi, Mengxing Huang

**Affiliations:** 1 College of Information and Communication Engineering, Hainan University, Haikou 570228, China; 20081000110003@hainanu.edu.cn (W.W.); charlieqi02@gmail.com (Z.Q.); 2 College of Electronic and Information Engineering, Guangdong Ocean University, Zhanjiang 524088, China; zhangqiaoxin@stu.gdou.edu.cn

**Keywords:** sonar image, shadow information, attention mechanism, lightweight, real-time detection, ocean monitoring

## Abstract

Sonar imaging technology is widely used in the field of marine and underwater monitoring because sound waves can be transmitted in elastic media, such as the atmosphere and seawater, without much interference. In underwater object detection, due to the unique characteristics of the monitored sonar image, and since the target in an image is often accompanied by its own shadow, we can use the relative relationship between the shadow and the target for detection. To make use of shadow-information-aided detection and realize accurate real-time detection in sonar images, we put forward a network based on a lightweight module. By using the attention mechanism with a global receptive field, the network can make the target pay attention to the shadow information in the global environment, and because of its exquisite design, the computational time of the network is greatly reduced. Specifically, we design a ShuffleBlock model adapted to Hourglass to make the backbone network lighter. The concept of CNN dimension reduction is applied to MHSA to make it more efficient while paying attention to global features. Finally, CenterNet’s unreasonable distribution method of positive and negative samples is improved. Simulation experiments were carried out using the proposed sonar object detection dataset. The experimental results further verify that our improved model has obvious advantages over many existing conventional deep learning models. Moreover, the real-time monitoring performance of our proposed model is more conducive to the implementation in the field of ocean monitoring.

## 1. Introduction

The optical environment under the sea surface is often uncertain. As a result of this unpredictable atmospheric environment, underwater images captured via infrared and visible imaging are often dim and unclear [1], which increases the difficulty of object detection and the positioning of unmanned underwater vehicles in bad weather, and seriously restricts the application of infrared and visible imaging technology in the field of ocean object detection. Since sound waves are mechanical waves that can travel through the elastic media of the atmosphere and seawater without much interference, sonar imaging technology has been widely used in the field of target monitoring and tracking at sea and underwater.

Although the underwater sound field environment is not greatly influenced by changes in the atmospheric environment, it will still be influenced by the underwater environment and hardware equipment, so the produced sonar image will have poor contrast and more noise, posing a great challenge to the accurate detection of sonar image targets. Therefore, with the development of digital image processing technology, many computer vision methods have been applied to sonar-related work [2,3,4,5], and object detection is no exception.

Most of the early machine vision processing methods first used image processing methods to extract the contours, textures, and other features of objects in sonar images, and then used machine learning or pattern matching methods to classify object features; some examples of these early methods include oriented gradient histogram (HOG) [6], support vector machine (SVM) [7], and singular value decomposition (SVD) [8]. Although these methods can achieve sonar image object detection, they generally have low detection accuracy and high algorithmic complexity.

In recent years, deep learning has been widely used in the field of machine vision due to its powerful feature learning ability, such as object detection [9] and object tracking [10]. Presently, neural networks based on deep learning, primarily ConvNet and Transformer [11], are predominantly used in general object detection. YOLOv3 [12] and Faster R-CNN [13] are representative ConvNets, primarily constructed out of convolutional layers. Typical transformers in machine vision include ViT [14] and Swin Transformer [15], which consist of encoders and decoders, and the self-attention block [11] within them possesses the global feature extraction. While ConvNets boast a simple structure and rapid detection speed, their detection accuracies are relatively low. Conversely, Transformer offers high detection accuracy but demands a significant amount of computational resources and a large number of parameters, along with a lengthy model training process. Inspired by these algorithms, numerous sonar object detection methods based on ConvNet and Transformer have been proposed in the field of sonar object detection.

For example, an attention-mechanism-based object detection method for a single-stage submarine is proposed in [16]. This method introduced an attention mechanism module to the CNN, and used the denoising module to suppress background noise interference. Zhen et al. [17] designed an adaptive global feature enhancement network based on a multi-scale receptive field feature extraction block and self-attention mechanism to solve the problem of noise affecting object detection in sonar images. Although the existing sonar object detection methods based on ConvNet have achieved good results, these methods face such problems as insufficient feature extraction, cumbersome calculation processes, slow detection speeds, and so on, hindering their capacity for the real-time detection and positioning of sonar targets. It is worth noting the work of Yongcan Yu et al. [18], who proposed a real-time automatic target recognition (ATR) method for underwater maritime object detection in side-scan sonar images based on Transformer-YOLOv5. This work demonstrates the potential of integrating advanced deep learning techniques, such as Transformer-YOLOv5, into sonar object detection methods to enhance their performance. However, the large parameters contained in Transformer-YOLOv5 is difficult to port to some machines.

Therefore, given the above problems, this paper designs a CenterNet-Saccade based on the CenterNet object detection model that can effectively extract global features. The proposed module is very lightweight and has some portability. With the addition of this module, the model’s detection accuracy and speed are greatly improved, surpassing the performance of the existing common ConvNet.

The main contributions of this study are as follows:

1. To capture and utilize the global environment information containing shadows in sonar images, a self-attention mechanism called E-MHSA is designed in ConvNet. E-MHSA can capture the global features in the environment and only consumes a little memory, namely GPU memory and computer RAM.
2. ShuffleBlock is designed as a block in the backbone Hourglass network, and greatly reduces consumption of computational resources. In addition, a hyperparameter γ is set for the selection of positive and negative samples to improve the detection accuracy of CenterNet.
3. Experiments show that our proposed model can effectively use parameters and calculations, and has faster reasoning speed and higher detection accuracy than the existing popular ConvNet algorithm.

## 2. Related Work

### 2.1. Anchor-Free

Recently, Anchor-Free has been shown to have powerful capabilities in computer vision, such as object detection, image classification, and image segmentation. Since it does not need to tune hyperparameters related to an anchor, it not only avoids a large number of IoU calculations between ground truth (GT) boxes and anchor boxes, but it also allows the training process to occupy less memory. For these reasons, Anchor-Free has attracted much attention.

Law et al. [19] proposed a method to detect targets by detecting the diagonal points of the prediction box, and also designed Corner Pooling to better locate the corner points of the prediction box, thus bypassing the anchor point problem; their method achieved the highest accuracy in one-SRAGE object detection. Tian et al. [20] classified and regressed anchor points, in which regression predicted the distance between anchor points and the four left and right boundaries above and below the detection box. At the same time, detection and other tasks using FCN were unified, allowing the convenient reuse of tasks such as semantic segmentation. Based on a backbone network with an encoder–decoder structure, Zhou et al. [21] proposed a simple and efficient, three-network structure for the output head of the model to output the predicted values. The three networks in the structure are, respectively, the category prediction heat map, the coordinates of two predicted center points, and the offset of the two center points. In addition, due to the large output resolution of the model, the recall effect is better for small targets.

To avoid tedious and time-consuming post-processing and to address the issue of multiple small targets being present in sonar images, we decided to use the CenterNet in Anchor-Free as the basic sonar detection model.

### 2.2. Lightweight Architecture

Real-world tasks are often designed with the aim of achieving optimal accuracy with limited computational budgets, target platforms (such as hardware), and application scenarios (for example, autonomous driving requires low latency). This has driven a number of works toward lightweight architecture designs and better speed–accuracy tradeoffs, including MobileNet [22], MobileNet V2 [23], ShuffleNet [24], and ShuffleNetV2 [25]. After considering the architecture of Hourglass [26] and the reality of underwater monitoring, we developed a lightweight Hourglass network based on the methods of MobileNet and ShuffleNetV2.

The specific method employed by MobileNet to reduce the number of network parameters is to decompose a complete convolution operation into two steps, namely depthwise convolution (DWConv for short) [27] and pointwise convolution. DWConv is different from conventional convolution where each convolution kernel operates in each channel of input data at the same time. In DWConv, each convolution kernel is responsible for only one channel. From the perspective of grouping convolution, it groups the input (output) channels into the corresponding number channels. Pointwise convolution performs convolution of a 1×1 convolution kernel size on depthwise results and associates the feature maps of N channels together.

ShuffleNetV2 uses four strategies to reduce network complexity: (1) The convolution layer consumes the least memory access cost (MAC) when the input and output channels are the same. (2) The number of grouped convolution operations is reduced appropriately. Although the grouped convolution operation will reduce the number of parameters, it will increase the consumption of MACs (multiply–accumulate operations), and the model inference speed will slow down. (3) The smaller the number of branches in the model, the lower the degree of network parallelism, and the faster the model speed. (4) The number of elementwise operations such as Add and Relu are reduced to reduce computing time. In our work, we sacrifice part of the ShuffleNetV2 strategy to fit the structure of Hourglass, and then use the MobileNet method to optimize Hourglass so that the accuracy and speed are excellent.

### 2.3. Self-Attention Modules

The attention mechanism enables a model to screen out a small amount of important information from a larger set and then focus attention on these important points of information, and it has been widely used in many tasks [28,29]. In particular, Ashish Vaswani et al. [11] proposed using a self-attention mechanism to extract the global dependence of the input and applied it to machine translation, and then proposed a multi-head attention mechanism based on this. As the function of the attention mechanism in the algorithm is to enable the model to screen out a small amount of important information from a larger and focus on this important information, the larger the weight, the more it focuses on the corresponding value. That is, the weight represents the importance of the information, and the value is the corresponding information. Self-attention mechanism are variations of attention mechanism that are better at capturing internal correlations of data or features. Auto-attention computes the correlation between a single feature query and all other feature keys and assigns it to value as a weight. The multi-head self-attention mechanism is an evolved version of the common single-head self-attention mechanism. It divides each attention operation into multiple heads and can extract feature information from multiple dimensions.

With the rising popularity of Transformer in recent years, self-attention mechanisms are increasingly being used as the core Transformer architecture, especially in the field of computer vision (CV). However, the huge memory and computing power required by the self-attention mechanism seriously limit the practical application of the corresponding CV work. In order to reduce the high memory and computation power requirements of self-attention, some researchers have tried to reduce the size of the input matrix. Classically, Dosovitskiy et al. cut the data into patches [14]. On this basis, Liu et al. [15] divided the input data into windows and restricted attention to windows with a smaller size, which greatly reduced the computational burden. Differently from works based on Transformer, our CNN-based model can solve the problem of self-attention MAC more concisely and efficiently, and make full use of the translation invariance of CNNs without adding additional embedding layers.

## 3. Method

### 3.1. Enrichment Multi-Head Self-Attention

Due to the translation invariance and scale invariance of CNNs, the receiving domain of each convolution kernel is quite limited, and the global feature information cannot be extracted, so it is difficult for the target to capture the shadow information in the global field of view. To allow the model to better capture the environmental features, including shadows, w e adopted and improved the multi-head self-attention (MHSA) mechanism.

The formula of self-attention is as follows:

(1) $$\mathrm{Attention}\left(Q,K,V\right)=\mathrm{softmax}\left(QK^{T}\right)V$$

where Q, K, and V refer to Query, Key, and Value, respectively, which are calculated from the same input data through different fully connected layers. After multiplying the transpose of Q by K, Softmax is used to calculate and normalize the weight map of V.

**Figure 1:**
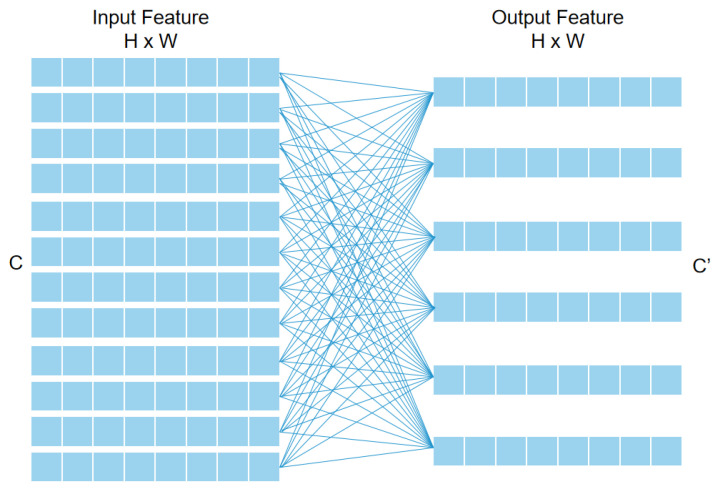
Fully connected computation in a self-attention mechanism.

Take Query as an example. Suppose that the input data take the form of a 3D matrix $X\in R^{B\times (H\times W)\times C}$ and the output data are a 3D matrix $X^{\prime }\in R^{B\times \left(H\times W\right)\times C^{\prime }}$ , where B is the batch size of the input data, H and W are the length and width of the data, C is the number of input channels, and C′ is the number of output channels. The calculation process is shown in Figure 1, and the number of parameters required for Query is $W\in R^{C\times C^{\prime }}$ . This means that a fully joined computational mapping from the input data to Query is equivalent to a 1×1 convolution computation on the input data. Therefore, we can replace this fully connected layer with a richer convolutional layer, such as a 3×3 convolutional layer.

In the process of our MHSA experiments, we found that when the H×W of Query, Key, and Value is unchanged and C′ increases, the memory access cost is not obvious. However, when the H×W of Query, Key, and Value increases and C′ remains the same, although the number of parameters decreases slightly, the memory access cost is quite large. Therefore, we believe that the main bulk of computation is in the calculation of Query, Key, and Value. If the convolutional layer is used to downsample the input data first to reduce H and W, then the MAC of the MHSA can be reasonable without losing information. Therefore, in this model, a 3×3 convolutional layer with stride 2 is used.

As shown in Table 1, compared with the method of QKV initialization using convolution with different kernel sizes, although the number of parameters generated by the 3×3 convolution is more than that of the 1×1 convolution, the QKV with a smaller size obtained after downsampling consumes much less memory and has a lower operation time in subsequent operations such as QK dot product. This not only saves valuable running memory, but it also does not slow down the original CenterNet model.

### 3.2. Lightweight Hourglass

**Figure 2:**
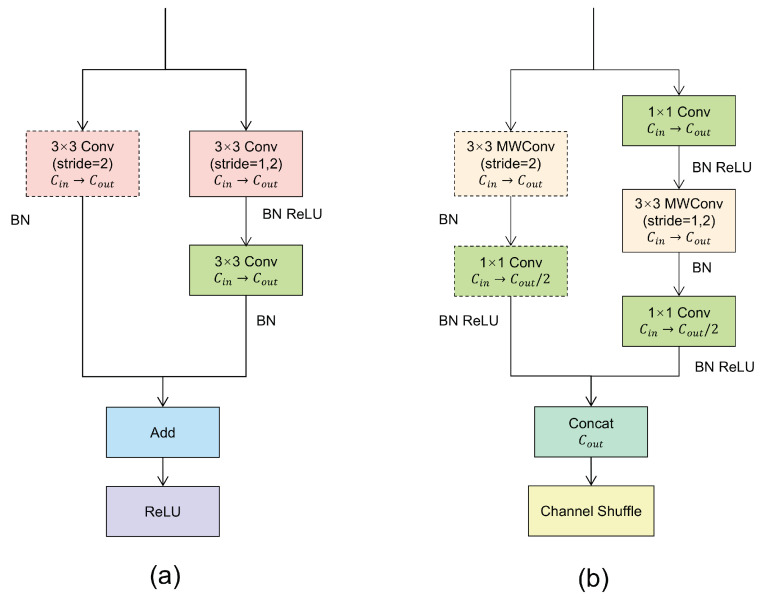
Residual blocks and building blocks for this work. (**a**) Basic residual element. (**b**) Improved ShuffleBlock. The dashed box on the left side of the module represents the operation on the skip layer during the current sampling, and there is no operation in the dashed box at other times.

The main computing resource set in CenterNet is Hourglass. Hourglass consists of the residual blocks shown in Figure 2a, two convolution layers of 3×3 , and a skip layer, which is replaced by a two 3×3 convolution layer when subsampling. Although Hourglass has a strong performance, the number of parameters and the reasoning time are too expensive. To reduce the calculation burden of Hourglass, the residual block is replaced by a 1×1 convolution layer and a depthwise separable convolution; the skip layer is also changed to depthwise separable convolution, and then Concat and channel shuffle functions are performed to exchange information between different channels, as shown in Figure 2b. Hereafter, the whole block is referred to as ShuffleBlock for short.

We have weighed the large number of channel transitions contained in Hourglass against the structure of ShuffleBlock, where channel transitions occur only after 3×3 convolution. In order for the matrix to correspond to the number of output channels, the number of channels should be converted to half the number of output channels before Concat is performed.

### 3.3. Adjusting Positive and Negative Samples

Although E-MHSA and L-Hourglass have improved the performance of CenterNet in different aspects, there is still more room for improvement. Through experiments, we found that adjusting the positive and negative samples can enhance the detection robustness of CenterNet. The following equation represents the loss function used by the original CenterNet:

(2) $$L_{k}=-\frac{1}{N}\sum_{\mathrm{xyc}}\left\{\begin{array}{cc} {\left(Y_{\mathrm{xyc}}\right)}^{\beta }{\left(1-{\hat{Y}}_{\mathrm{xyc}}\right)}^{\alpha }\log\left({\hat{Y}}_{\mathrm{xyc}}\right)\; & \mathrm{if}\;Y_{\mathrm{xyc}}=1\\ {\left(1-Y_{\mathrm{xyc}}\right)}^{\beta }{\left({\hat{Y}}_{\mathrm{xyc}}\right)}^{\alpha }\log\left(1-{\hat{Y}}_{\mathrm{xyc}}\right)\; & \mathrm{otherwise} \end{array}\right.$$

where α and β are adjustable focus parameters, and ${\left({\hat{Y}}_{\mathrm{xyc}}\right)}^{\alpha }$ and ${\left(1-{\hat{Y}}_{\mathrm{xyc}}\right)}^{\alpha }$ are adjusted to address the issue of the gradient being dominated by easy examples. ${\left(Y_{\mathrm{xyc}}\right)}^{\beta }$ and ${\left(1-Y_{\mathrm{xyc}}\right)}^{\beta }$ are used to adjust the weight of positive and negative samples in the loss. However, the loss Function (2) fundamentally still assigns only one positive sample to each target, while the rest are considered negative samples, resulting in a severe imbalance between positive and negative samples. To obtain more useful positive samples, we introduce a hyperparameter γ in the calculation of the classification loss, resulting in the following modified loss function:

(3) $$L_{k}=-\frac{1}{N}\sum_{\mathrm{xyc}}\left\{\begin{array}{cc} {\left(Y_{\mathrm{xyc}}\right)}^{\beta }{\left(1-{\hat{Y}}_{\mathrm{xyc}}\right)}^{\alpha }\log\left({\hat{Y}}_{\mathrm{xyc}}\right)\; & \mathrm{if}\;Y_{\mathrm{xyc}}\geq \gamma \\ {\left(1-Y_{\mathrm{xyc}}\right)}^{\beta }{\left({\hat{Y}}_{\mathrm{xyc}}\right)}^{\alpha }\log\left(1-{\hat{Y}}_{\mathrm{xyc}}\right)\; & \mathrm{otherwise} \end{array}\right.$$

We divide the pixels whose label values are greater than the threshold γ into positive samples and take their label values as the corresponding weight of the loss function. When γ=1 , that is, when a target corresponds to only one positive sample with a label value of 1, it is the original CenterNet setting.

## 4. Experiments and Analysis

To evaluate the proposed method, we conduct comprehensive experiments on underwater forward-looking sonar datasets provided by Pengcheng Laboratory. The experimental results show that, taking PASCALVOC2012 as the evaluation standard, our model achieves good performance when the IoU threshold is 0.5–0.8. In the following subsections, we first present the details of the dataset and experimental implementation, and then we present the experimental results of a series of ablation experiments and comparison experiments performed on the forward-looking underwater sonar dataset.

### 4.1. Sonar Image Dataset and Experimental Details

Our experiment is based on the acoustic image dataset launched by the Pengcheng Laboratory, which uses a Tritech Gemini 1200I multi-beam forward-looking sonar as the data acquisition device. The images are stored in BMP format. There are a total of 5000 images, including 3200 images in the training set, 800 images in the validation set, and 1000 images in the test set.

The model train and test environments in this paper are Linux Ubuntu version 16.04LTS, running on Inter-core I9-9900K, with a TITAN RTX and 31.3 GB of memory. To ensure the stability and reproducibility of the experiment, we chose the version that is most compatible with the hardware, namely the programming environments of Python 1.7.0, CUDA 10.2, and CUDNN 7.6.532, which will ensure the validity of our research results. After several experiments and comprehensive consideration, we adopt the following settings to train the sonar dataset from scratch: the size of input resolution is fixed as 512×512 , the optimizer uses Adam, the basic learning rate is set as 0.0001, the number of training rounds is 150, the training strategy of learning rate fixed step size decrease is adopted, each decrease is 1/10 of the original, and the number of decreasing rounds is 80 and 110, respectively. To enhance the diversity of data, we used random vertical flipping and random horizontal flipping to preprocess them. In addition, the classic algorithms, such as SSD, used in the comparative experiments are based on the open-source framework MMDetection. These experiments use the same sonar image dataset, and their evaluation metric is Pascal VOC2012 standard, which calculates the Average Precision (AP) by averaging the accuracy values at all different recall points.

### 4.2. Visualized E-MHSA

**Figure 3:**
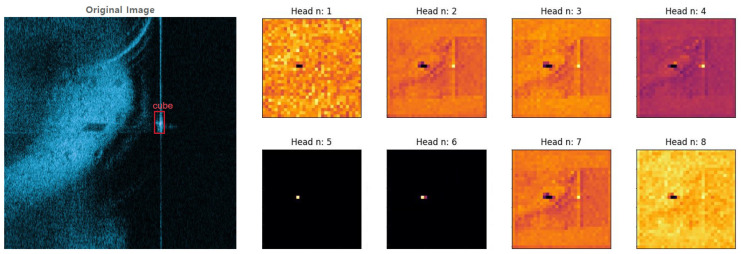
On the left is the original image, and on the right are eight heat maps corresponding to the outputs of the eight different heads of E-MHSA.

Before formally starting the MHSA experiments, to validate the effectiveness of the MHSA, we visualized E-MHSA and created heat maps, as shown in Figure 3. From these heat maps, we can see that E-MHSA, in addition to focusing on the target, also prominently attends to the shadows of the target, effectively distinguishing the shadows from the surrounding environment.

### 4.3. MHSA Experimentation

In order to compare the influence of different QKV initialization methods on the detection accuracy, number of parameters, and running speed of CenterNet with similar MACs, we conducted the experiments shown Table 2 and also generated heat maps and inspection renderings to visualize some of the sonar images. It can be seen that using 3×3 convolution as the object of attention during QKV initialization prediction is more comprehensive and accurate; it is also faster.

### 4.4. Ablation Study

To verify the influence of different positive sample partition parameters γ on CenterNet-Saccade, we set up ablation experiments as shown in Table 3. Under the PASCAL VOC2012 standard, the verification IoU thresholds are 0.5, 0.6, 0.7, and 0.8. We can see that different values of γ have different effects on different IoU threshold verification scores.

### 4.5. Comparative Experiments

Our model is compared with other classical object detection models on the same underwater forward-looking sonar dataset, as shown in Table 4, which shows the average accuracy of each model under different IoU thresholds and using the PASCAL VOC2012 evaluation standard. From the last four experiments, it can be observed that the model’s scores significantly improved after adding E-MHSA, but the computation time also increased noticeably. After replacing the network with a lightweight Hourglass, CenterNet achieved good scores with a short computation time and little RAM consumption.

In Table 5, the accuracy of our model and other classical object detection models is compared in eight categories when IoU = 0.7. It can be seen that the detection performance of our model is the best in each category.

## 5. Conclusions

In this study, a new sonar image object detection algorithm called CenterNet-Saccade is proposed. This method can effectively extract and use the global semantic features, including the target object’s shadow, in sonar images to achieve the accurate detection of different types of sonar targets. For CenterNet-Saccade, we designed an evaporated MHSA to capture global information so that background information such as the target’s shadow features could be obtained while maintaining a small MAC and enhancing the correlation between target features and shadow features. ShuffleBlock greatly reduces the number of parameters and MAC of Hourglass, thus improving the detection speed of the model. In addition, a hyperparameter γ is added to the selection of positive and negative samples to further improve its detection accuracy. The qualitative and quantitative experimental results show that CenterNet-Saccade has higher detection accuracy and faster detection speed. In addition, an aspect of the CenterNet-Saccade algorithm still worth exploring regards transferring the algorithm to real-world robots. How to optimize the structure of the algorithm to enable real-time data processing and seamless integration into autonomous robots for practical applications will be studied in the future.

## Figures and Tables

**Table 1:** Memory, running time, and Params corresponding to different formulas; 64×64→64×64 means that 1×1 convolution is used to preserve the dimension of the input matrix, 64×64→32×32 means that 3×3 convolution is used to down-sample the matrix, Dot denotes the Dot product, and $\mathrm{Attn}=\mathrm{softmax}(QK^{T})$ .

H × W	Formular	Params	MAC	Time (s/10^3^)
64×64→64×64	QKV Initialization	196,608	269,549,824	21.01
	Dot (Q, K^T^)	0	136,314,880	252.48
64×64	Softmax	0	402,685,952	462.64
	Dot (Attn, V^T^)	0	136,314,880	295.53
64×64→32×32	QKV Initialization	1,769,472	2,416,771,328	28.01
	Dot (Q, K^T^)	0	8,912,896	13.00
32×32	Softmax	0	25,174,016	24.01
	Dot (Attn, V^T^)	0	8,912,896	73.01

**Table 2:** Comparison of accuracy (IoU threshold = 0.7), parameter number, and speed based on original CenterNet for different MHSA methods.

Method	${\mathrm{AP}}_{70}^{\mathrm{val}}$ (%)	Number of Params	Time (s/10^3^)
Base ( 1×256×32×32 )	75.77	4,431,360	109.30
Grouping of FNN ( 1×256×32×32 )	75.53	14,592	109.04
Conv 1×1 ( 1×256×32×32 )	75.70	4,431,360	90.33
Conv 3×3 ( 1×384×64×64 )	75.95	1,769,984	34.44

**Table 3:** Under the PASCAL VOC2012 standard, each parameter γ sample is divided in different IoU thresholds to determine average precision.

γ	${\mathrm{AP}}_{50}^{\mathrm{val}}$ (%)	${\mathrm{AP}}_{60}^{\mathrm{val}}$ (%)	${\mathrm{AP}}_{70}^{\mathrm{val}}$ (%)	${\mathrm{AP}}_{80}^{\mathrm{val}}$ (%)
1.00	98.45	92.83	74.25	24.55
0.90	98.11	94.71	78.86	26.96
0.95	98.31	93.47	76.84	23.27
0.80	98.06	94.53	74.93	25.11
0.70	97.83	93.45	73.81	25.50

**Table 4:** The average accuracy of different IoUs under the PASCAL VOC2012 standard, inference time, and memory cost for each model.

Methods	${\mathrm{AP}}_{50}^{\mathrm{val}}$ (%)	${\mathrm{AP}}_{60}^{\mathrm{val}}$ (%)	${\mathrm{AP}}_{70}^{\mathrm{val}}$ (%)	${\mathrm{AP}}_{80}^{\mathrm{val}}$ (%)	Time (s)	Mem (GB)
YOLOv3	86.5	79.0	56.5	20.7	0.311	2.7
SSD [30]	88.4	82.9	68.8	23.4	0.361	9.3
RetinaNet [31]	90.8	86.5	69.8	25.1	0.387	3.7
FCOS	92.2	86.9	69.3	24.5	0.494	3.5
Faster R-CNN	89.1	86.0	71.1	26.2	0.445	3.9
CenterNet	98.5	92.8	72.1	24.6	0.376	3.3
CenterNet (E-MHSA)	95.6	94.8	77.3	28.8	0.600	3.5
CenterNet (L-Hourglass)	98.3	90.2	71.4	23.9	0.102	1.4
CenterNet-Saccade	97.7	94.2	76.5	26.7	0.156	1.7

**Table 5:** The precision of each model category with an IoU threshold of 0.7 under PASCAL VOC2012 standard.

Methods	${\mathrm{AP}}_{70}^{\mathrm{val}}$ (%)	Ball (%)	Cylindb Aller (%)	Square Cage (%)	Cube (%)	Circle Cage (%)	Human Body (%)	Metal Bucket (%)	Tyre (%)
YOLOv3	56.5	72.9	50.7	53.0	63.7	56.6	51.0	51.5	52.9
SSD	68.8	75.2	65.4	59.4	72.9	74.2	60.0	74.7	69.0
RetinaNet	69.8	72.7	66.2	59.0	77.7	74.6	67.5	73.2	68.8
FCOS	69.3	74.5	65.4	62.2	76.4	73.9	68.1	73.5	68.4
Faster R-CNN	71.1	75.8	61.8	67.0	76.1	77.4	71.6	76.5	62.7
CenterNet	74.3	78.0	71.6	57.1	83.6	72.0	73.3	78.3	63.3
CenterNet-Saccade	76.5	79.6	75.9	66.3	85.0	74.1	81.1	74.0	75.9

## Data Availability

The code and dataset used and analyzed in the current research can be obtained from the following link upon reasonable request: Code: https://github.com/will319/centernet-saccade (accessed on 4 December 2023). Dataset: https://pan.baidu.com/s/1n2868mAKC2pZ1Efy_oc1kQ?pwd=ctsc (accessed on 4 December 2023). If the link fails, you can contact the corresponding author to obtain a new link.
